# Christopher Elliott

**Published:** 1933

**Authors:** 


### CHRISTOPHER ELLIOTT, M.D.

Of late years Dr. Christopher Elliott, who passed away on the 14th of February, would have been known only to a few Bristol practitioners. He had been out of active practice for a good many years, and was greatly handicapped by deafness. Not long ago he showed grave symptoms of heart weakness, and it looked as if his days were numbered, but he pulled through, got about actively, and outlived his medical adviser.

For many years he was Physician to the Children's Hospital, and he carried on an extensive practice in Pembroke Road, Clifton. But it is doubtful if medicine was ever his main interest in life ; certainly not in his later years. He was widely read, and liked to talk of things far beyond the bounds of his profession. But his heart was principally set upon religious and philanthropic work. He did a good deal of preaching in various places of worship in the town. He was Chairman of Trustees of the Orphanage on Ashley Down founded nearly a hundred years ago by George Müller. For forty-seven years he was Treasurer of the Bristol Institute and Mission for the Deaf and Dumb, and not the least impressive of the funeral tributes paid to his memory was by a very large congregation of deaf-mutes at 4 King Square, conducted in sign language.

The medical profession finds room within its ranks for men of many types, and it is well that it is so.

